# Psychological associations between perceived generative AI-supported learning and creative design performance in vocational design education: a within-course correlational study

**DOI:** 10.3389/fpsyg.2026.1962146

**Published:** 2026-10-07

**Authors:** Xiaoping Xu, Chenhui Wang, Shuangyan Fu, Jiawei Tong, Dian Jin

**Affiliations:** 1 Design College, Jinhua University of Vocational Technology, Jinhua, China; 2 Faculty of Design and Architecture, Universiti Putra Malaysia, Serdang, Selangor, Malaysia; 3 College of Design and Innovation, Zhejiang Normal University, Jinhua, China; 4 Psychological Counseling Center, Jinhua University of Vocational Technology, Jinhua, China; 5 College of Education, Wenzhou-Kean University, Wenzhou, China

**Keywords:** creative design performance, creative self-efficacy, sustainable design cognition, generative artificial intelligence, learning engagement, vocational design education

## Abstract

Generative artificial intelligence (GenAI) is increasingly used in design courses, but evidence remains limited on how students’ appraisal of such support co-varies with domain cognition, creative confidence, engagement, and independently assessed creative work. This within-course correlational study examined within-course change and theory-specified contemporaneous associations in a 72-h Furniture Appreciation and Selection course involving 150 vocational design students in China. Sustainable design cognition and creative self-efficacy were measured before and after the course; perceived GenAI-supported learning and learning engagement were measured after it; and final creative design performance was rated independently by three experts. Paired-samples t tests described within-course change. Item-level CFA preceded the score-level association analyses, while standardized composite-score ordinary least squares models and 10,000-resample percentile bootstrap confidence intervals summarized theory-specified post-course associations and coefficient products. Sustainable design cognition increased from 3.326 to 3.792, t (149) = 10.362, *p* < 0.001, d_z_ = 0.846, and creative self-efficacy increased from 3.509 to 3.877, t (149) = 7.424, *p* < 0.001, d_z_ = 0.606; these values indicate change during the course as a whole rather than a GenAI-specific intervention effect. Among the direct post-course associations, learning engagement showed the largest coefficient with expert-rated creative design performance (β = 0.515, *p* < 0.001; f^2^ = 0.361). The theory-specified coefficient products were 0.076 for SDC-CSE-LE, 0.204 for CSE-LE-CP, and 0.0080 for AISL-SDC-CSE-LE-CP. The last interval narrowly excluded zero [0.0002, 0.0234], but the product was statistically detectable and negligible in magnitude. Because the study followed one cohort without a comparison condition and most model variables were measured at the same post-course occasion, the findings support within-course change and contemporaneous association, not causal or temporally ordered effects.

## 1 Introduction

Generative artificial intelligence (GenAI) has moved quickly into design classrooms. Text- and image-generation systems can externalize ideas, produce alternatives, and lower technical barriers to visualization. These affordances are useful, but they do not show whether students think more carefully, persist through revision, or make better design decisions. The educational question is therefore how perceived GenAI support relates to the cognitive, motivational, and behavioral processes associated with a completed creative outcome.

Recent research presents a mixed picture. Broader reviews emphasize that GenAI offers rapid feedback and ideation support but also creates risks of overreliance, inaccuracy, and uncritical adoption, making AI literacy central to responsible educational use (Kasneci et al., 2023; Ng et al., 2021; Tlili et al., 2023). Creativity research likewise shows both opportunity and constraint: GenAI can support divergent ideation and individual creative output, yet benefits depend on how the tool is used and may coincide with reduced diversity across outputs (Haase and Hanel, 2023; Habib et al., 2024; Ivcevic and Grandinetti, 2024; Doshi and Hauser, 2024). Hwang and Wu (2025) linked AI-related perceptions with design students’ creative cognition through self-efficacy and reduced anxiety. Song and Xu (2026) reported stronger outcomes when GenAI-supported design learning was organized through a structured design-thinking strategy. Recent design-specific studies similarly emphasize structured, human-led integration: Hatef Jalil and Abdullah (2026) developed an AI-integrated design-thinking model for vocational fashion design, while Balasubramanian (2026) described curriculum implementation in fashion design that treated GenAI as a supporting tool rather than a replacement for human creative judgment. Other findings point to limits: AI dependency may undermine motivation through learned helplessness (Xiaohui and Khan, 2026), while stronger perceived interaction was associated with greater perceived cognitive burden in that context (Huang et al., 2026). Students also differ in AI literacy and cognitive flexibility, with corresponding differences in deep learning (Simo and Lina, 2026). GenAI, in short, is not a uniform instructional treatment; its value depends on how students understand and use the support available to them.

This issue is particularly relevant in vocational design education, where a creative proposal must be original, workable, and clearly justified. Furniture appreciation and selection requires students to interpret style, materials, structure, spatial fit, user needs, and aesthetic-functional quality. GenAI can expand the pool of references and possible solutions, but it cannot take responsibility for comparison, revision, or final judgment. Those tasks remain part of students’ disciplinary learning.

Sustainable design cognition adds a domain-specific requirement. Design students are increasingly expected to consider material responsibility, durability, repairability, resource use, health, comfort, and long-term value (Ceschin and Gaziulusoy, 2016; Faludi et al., 2023; Watkins et al., 2021). Generated images may make alternatives easier to see, but visual fluency is not the same as sustainable judgment. Students still have to apply criteria, reject superficial solutions, and explain the consequences of their choices.

Social cognitive theory helps separate these elements. In the present model, perceived GenAI-supported learning represents an appraisal of the learning environment; sustainable design cognition represents domain understanding; creative self-efficacy represents confidence in creative capability; learning engagement represents cognitive, emotional, and behavioral participation; and creative design performance represents the quality of completed work. This separation prevents perceived technological usefulness from being treated as creative design performance. The outcome was instead rated by experts, consistent with the consensual assessment tradition in creativity research (Amabile, 1982).

Three unresolved issues guided the study. Sustainable design cognition has rarely been examined as a domain-specific psychological resource in GenAI-supported design learning. Much of the available evidence relies on perceived usefulness, intention, or self-reported creativity rather than independently evaluated work. It is also unclear whether a long theory-specified sequence of post-course associations carries substantive empirical weight, or whether relationships closer to active engagement and completed work are stronger. Addressing these issues connects the study directly to cognitive mechanisms, learning outcomes, and evidence-based practice while avoiding the assumption that a contemporaneous association model can establish a temporal psychological pathway.

The study therefore combined matched pre-course and post-course measures, a theory-specified contemporaneous post-course association model, and ratings of final work by three experts. It asked: (RQ1) How did sustainable design cognition and creative self-efficacy change during the course? (RQ2) How were perceived GenAI-supported learning, sustainable design cognition, creative self-efficacy, learning engagement, and expert-rated creative design performance associated after the course? (RQ3) Which theory-specified coefficient products were statistically detectable, and how large were they in substantive terms?

## 2 Theoretical framework and research hypotheses

### 2.1 Social cognitive perspective

Social cognitive theory explains learning through reciprocal relations among environmental conditions, personal factors, behavior, and outcomes (Bandura, 1986). An educational resource does not determine performance by itself. Its relevance depends partly on how learners interpret the resource, judge their own capability, invest effort, and respond to feedback.

The proposed model follows this logic while distinguishing five constructs. Perceived GenAI-supported learning is an appraisal of environmental support; sustainable design cognition is domain-specific understanding; creative self-efficacy is a motivational belief; learning engagement is active participation; and creative design performance is an expert-rated outcome. The role assigned to efficacy beliefs is consistent with their established links to effort, persistence, and engagement (Bandura, 1997).

The model is deliberately narrower than the full reciprocal system described by social cognitive theory. Its arrows specify a theory-guided analytical order for organizing the associations; they do not establish temporal precedence, mediation, or causal direction. AISL, SDC, CSE, and LE were assessed at the same post-course occasion, so reverse and reciprocal explanations remain plausible. For example, sustained engagement may strengthen creative self-efficacy, and students with greater creative confidence or stronger task success may retrospectively appraise GenAI support more positively. The framework therefore treats social cognitive theory as an organizing rationale for construct separation rather than as evidence that the observed variables unfolded in a verified sequence.

### 2.2 Perceived GenAI-supported learning, sustainable design cognition, and creative self-efficacy

Perceived GenAI-supported learning refers to students’ judgment that GenAI tools assisted their understanding, ideation, comparison, expression, and independent thinking during the course. It is not a measure of use frequency or exposure to a standardized tool. Students can report similar perceived support while using different prompts, evaluation strategies, and levels of reliance. The construct is therefore interpreted as an appraisal of learning support, not as an experimental dose of technology.

In furniture learning, GenAI can visualize styles, materials, spatial arrangements, and alternative design atmospheres. Such representations may help students compare proposals against sustainability criteria, including durability, reuse, repairability, resource conservation, health, comfort, and long-term value. Students who perceive this process as supportive may also report clearer sustainable design understanding. This expected association concerns post-course scores; it does not assume that GenAI exposure independently produced the observed cognition.

GenAI-assisted ideation may also be related to creative self-efficacy. Rapid externalization and revision can make progress easier to observe, especially when students are working through unfamiliar problems. Previous studies have linked GenAI-supported design learning with creative self-efficacy and have shown that outcomes depend on the instructional structure surrounding the technology (Huang et al., 2024; Hwang and Wu, 2025; Song and Xu, 2026). Students who regard GenAI as a useful learning resource may therefore express stronger confidence in developing creative solutions. On this basis:

*H1*: Perceived GenAI-supported learning is positively associated with post-course sustainable design cognition.

*H2*: Perceived GenAI-supported learning is positively associated with post-course creative self-efficacy.

### 2.3 Sustainable design cognition and creative self-efficacy

Sustainable design cognition refers to students’ understanding of sustainability principles and their ability to use those principles when appreciating, selecting, and developing furniture solutions. Design for sustainability now encompasses circularity, systems thinking, social responsibility, and long-term value, rather than only environmental improvements to individual products (Ceschin and Gaziulusoy, 2016; Faludi et al., 2023). In furniture and interior-related education, it includes materials, durability, resource use, health, comfort, reuse, repairability, and life-cycle considerations (Afacan, 2014; Al-Saud et al., 2024; Chellappa and Rohatgi, 2025).

This cognition is not creative capability itself, but it can give students a firmer basis for making creative decisions. Clear sustainability criteria help them interpret requirements, evaluate GenAI-generated alternatives, and defend a final choice on more than visual appeal. In social cognitive terms, students’ judgments of capability are informed partly by how they interpret their knowledge and progress in a task (Bandura, 1997). A positive association between sustainable design cognition and creative self-efficacy was therefore expected:

*H3*: Post-course sustainable design cognition is positively associated with post-course creative self-efficacy.

### 2.4 Creative self-efficacy and learning engagement

Creative self-efficacy is the belief that one can produce creative outcomes (Tierney and Farmer, 2002). It is a motivational judgment, not a direct measure of creative ability. Students with stronger creative self-efficacy are generally more willing to approach uncertain tasks and persist when an initial idea is incomplete or unsuccessful (Beghetto, 2006; Mathisen and Brønnick, 2009).

Learning engagement concerns cognitive, emotional, behavioral, and agentic involvement in learning (Fredricks et al., 2004; Kahu, 2013; Reeve and Tseng, 2011). In this course, it included analyzing requirements, sustaining interest, participating in activities, comparing alternatives, seeking feedback, and revising proposals. Social cognitive theory links efficacy beliefs with task choice, effort, persistence, and responses to difficulty (Bandura, 1997). Creative confidence should therefore be positively associated with engagement in an iterative design course:

*H4*: Post-course creative self-efficacy is positively associated with post-course learning engagement.

### 2.5 Learning engagement and creative design performance

Creative design performance was defined as the quality of completed furniture selection and design work judged by experts. The assessment covered originality and effectiveness, the two elements in the standard definition of creativity (Runco and Jaeger, 2012). Effectiveness was represented by usefulness, aesthetic quality, spatial suitability, sustainability, technical presentation, and overall design quality. Expert ratings supplied an outcome source distinct from students’ reports of support, cognition, confidence, and engagement (Amabile, 1982).

A strong final proposal requires more than an initial concept. Students must interpret the problem, examine materials and spatial relationships, compare alternatives, respond to feedback, resolve inconsistencies, and refine the presentation. Engagement reflects participation in these activities and should therefore be associated with the quality of the completed work. Course-based research has likewise reported a positive relationship between learning engagement and creativity performance (Qian et al., 2026).

*H5*: Post-course learning engagement is positively associated with expert-rated creative design performance.

### 2.6 Theory-specified coefficient products

The five direct hypotheses define three theory-specified products of standardized coefficients. These products summarize whether the observed post-course associations are statistically compatible with the ordering shown in Figure 1. Because the constituent questionnaire constructs were measured at the same post-course occasion, the products are not treated as causal mediation effects or as evidence that one construct temporally preceded another.

**Figure 1:**
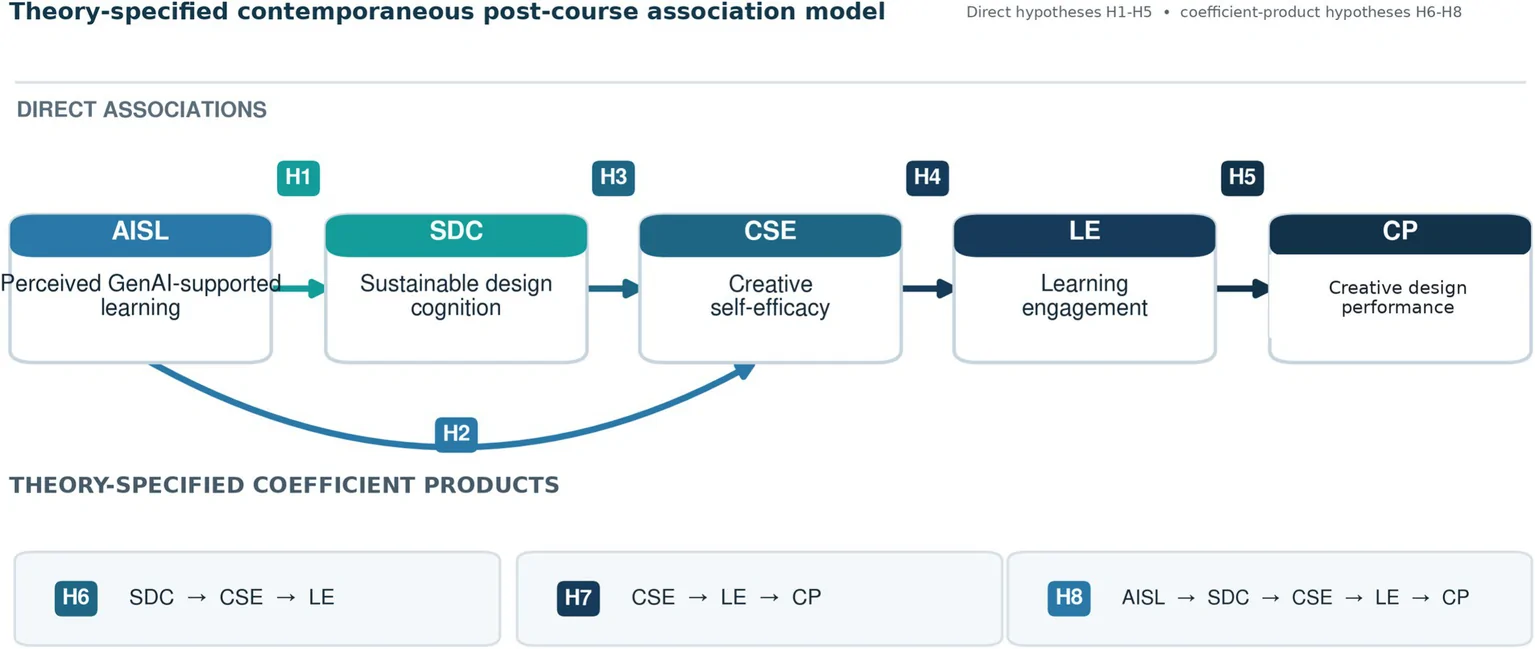
Theory-specified contemporaneous post-course association model. AISL, perceived GenAI-supported learning; SDC, sustainable design cognition; CSE, creative self-efficacy; LE, learning engagement; CP, expert-rated creative design performance. AISL, SDC, CSE, and LE were assessed at the same post-course occasion. Arrows indicate analytical ordering only and do not establish temporal precedence, mediation, or causal direction.

The first coefficient product combines the SDC-CSE and CSE-LE associations. Domain knowledge can provide criteria for evaluating design decisions, while creative self-efficacy reflects confidence in acting on those criteria. The hypothesis therefore concerns whether the product of these two theory-specified contemporaneous coefficients is positive, not whether CSE has been established as a temporal mediator.

*H6*: The theory-specified product of the post-course SDC-CSE and CSE-LE coefficients is positive.

The second coefficient product combines the CSE-LE and LE-CP associations. Creative confidence is not itself a completed design outcome, and engagement reflects participation in analysis, comparison, feedback, and revision activities that are more proximal to completed work. The hypothesis therefore concerns the sign and magnitude of the specified coefficient product.

*H7*: The theory-specified product of the post-course CSE-LE and LE-CP coefficients is positive.

The third coefficient product combines four contemporaneous associations: AISL-SDC, SDC-CSE, CSE-LE, and LE-CP. Because four coefficients are multiplied, the resulting value is expected to be substantially smaller than the component associations and must be interpreted by magnitude as well as by whether its bootstrap interval excludes zero. This is a descriptive test of compatibility with the proposed ordering, not a test of a causal serial mechanism.

*H8*: The theory-specified product of the post-course AISL-SDC, SDC-CSE, CSE-LE, and LE-CP coefficients is positive.

Figure 1 summarizes the theory-specified analytical ordering; the estimated direct associations and coefficient products are reported in Table 1 and Figure 2.

**Table 1:** Direct associations and theory-specified coefficient products from the post-course score-level analysis.

Hypothesis	Specified path	β/estimate	SE	t/95% CI	*p*	f^2^	Interpretation
H1	AISL → SDC	0.204	0.080	2.538	0.012	0.044	Positive; small local effect
H2	AISL → CSE	0.298	0.078	3.831	< 0.001	0.100	Positive; modest local effect
H3	SDC → CSE	0.192	0.078	2.464	0.015	0.041	Positive; small local effect
H4	CSE → LE	0.396	0.075	5.252	< 0.001	0.186	Positive; moderate local effect
H5	LE → CP	0.515	0.070	7.310	< 0.001	0.361	Positive; large local effect
Theory-specified coefficient products (10,000 percentile bootstrap resamples)							
H6	SDC → CSE → LE	0.076	0.037	[0.0114, 0.1574]	—	—	CI excludes 0; positive product
H7	CSE → LE → CP	0.204	0.051	[0.1095, 0.3089]	—	—	CI excludes 0; larger product
H8	AISL → SDC → CSE → LE → CP	0.0080	0.0060	[0.0002, 0.0234]	—	—	CI narrowly excludes 0; negligible

Direct associations report standardized OLS coefficients (β), analytic SE, t, *p*, and local f^2^. Outcome R^2^: SDC = 0.042; CSE = 0.149; LE = 0.157; CP = 0.265. Coefficient products report standardized estimates, bootstrap SE, and percentile 95% CIs from 10,000 resamples. An interval excluding zero is described as statistically detectable; magnitude is interpreted separately. Arrows denote theory-specified contemporaneous associations, not temporal or causal effects.

**Figure 2:**
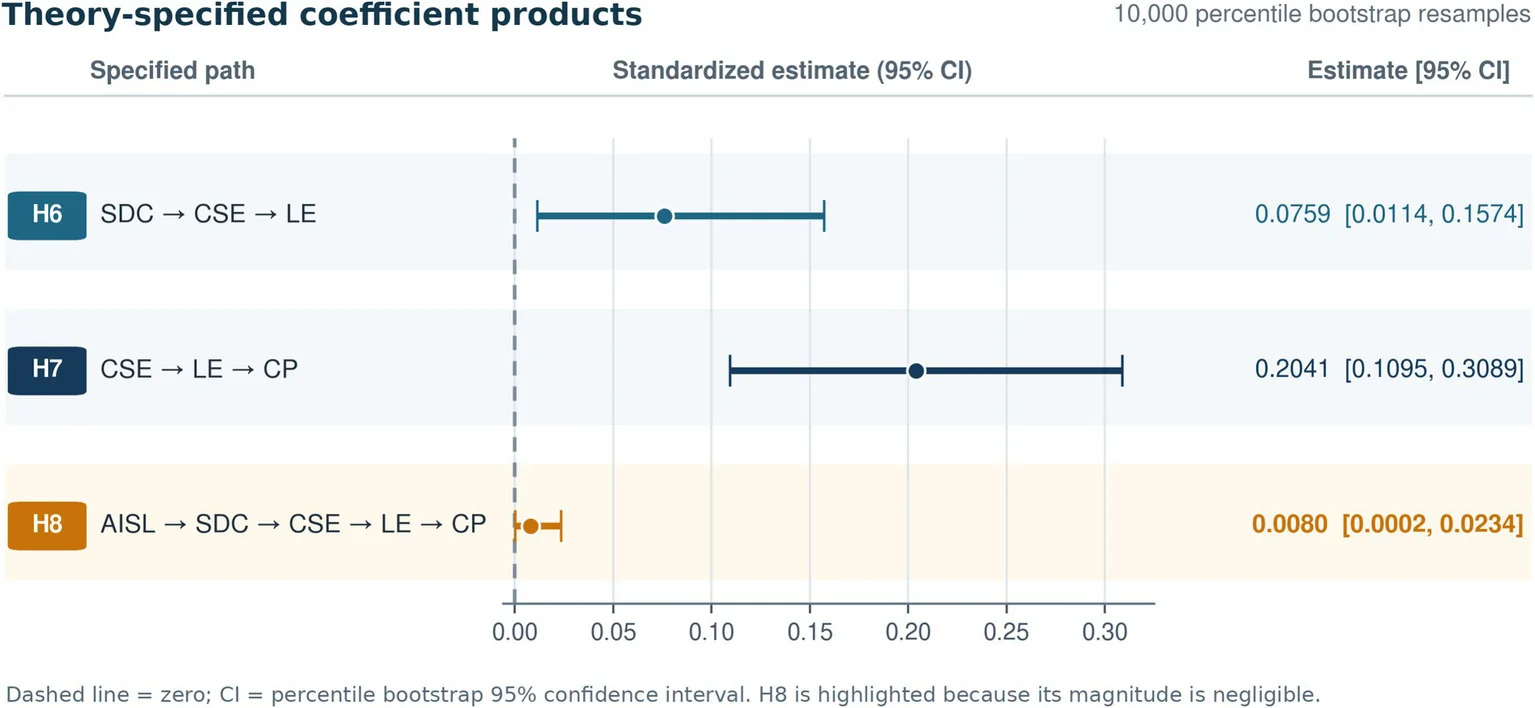
Bootstrap estimates and percentile 95% confidence intervals for theory-specified coefficient products. H8 narrowly excluded zero at unrounded precision but was negligible in magnitude. The products do not establish temporal mediation.

## 3 Materials and methods

### 3.1 Study design

A single-group pre-post course-based design was used for two analytically distinct purposes. The first analysis stream described within-course change in sustainable design cognition and creative self-efficacy across the 72-h Furniture Appreciation and Selection course. The second analysis stream examined contemporaneous post-course associations among perceived GenAI-supported learning, sustainable design cognition, creative self-efficacy, learning engagement, and expert-rated creative design performance. The pre-post stream is not used to identify a GenAI-specific intervention effect, and the post-course stream is not used to establish temporal mediation. Without a comparison condition, both streams are interpreted within the evidential limits of one observed course cohort.

The pre-course questionnaire measured sustainable design cognition and creative self-efficacy. After the course, students reported perceived GenAI-supported learning, sustainable design cognition, creative self-efficacy, and learning engagement. Three experts then rated the final furniture selection and design work. Combining student reports with an independently rated outcome reduced reliance on learning perceptions alone. Additional details on the course design, complete construct items and expert-rating criteria, theory-specified hypotheses, and additional diagnostic analyses are provided in Supplementary Table S1–S4.

### 3.2 Course design

The instructional activities were embedded in Furniture Appreciation and Selection, a practice-oriented course for vocational design students. The course connected furniture appreciation with design judgment and selection decisions through style recognition, material and structural analysis, spatial configuration, sustainability criteria, GenAI-supported concept development, and final presentation.

Sustainability was embedded as a core component of the course because design for sustainability has expanded from product-level environmental improvement to broader product-service, socio-spatial, and socio-technical approaches (Ceschin and Gaziulusoy, 2016). Recent design education research has also emphasized that sustainability should be treated as a foundational component of design curricula (Faludi et al., 2023). In furniture learning, students need to consider not only visual style and function, but also material selection, durability, repairability, resource conservation, health, comfort, and long-term use value.

The course proceeded through six stages: style recognition; material, craftsmanship, and structural analysis; furniture selection and spatial configuration; sustainable design cognition; GenAI-supported concept generation; and final presentation and assessment. Students used generative text and image tools, including ChatGPT, Midjourney, and Stable Diffusion, according to task needs; no single model or version was required or recorded at the participant level. Generated material served as a reference for comparison, ideation, and visualization, not as a final submission. Students retained prompts, compared alternatives, revised generated content, and justified their decisions. These artifacts documented the learning process but were not coded as research variables. Figure 3 summarizes the six instructional stages, learning activities, GenAI roles, and required outputs; no individual student’s work is reproduced.

**Figure 3:**
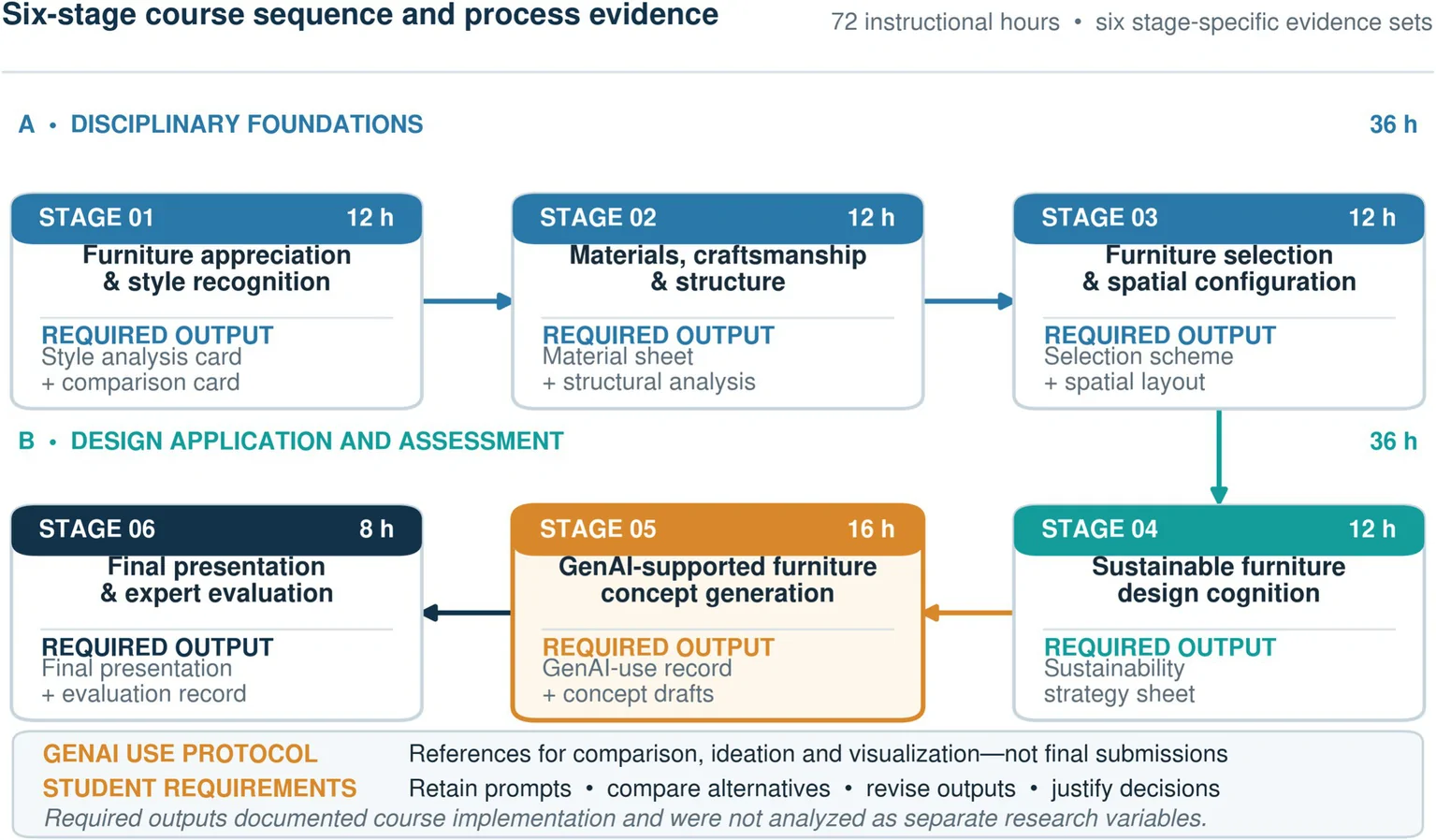
**(A)** Disciplinary foundations; **(B)** Design application and assessment. Figure 5, **(A)** Paired score distributions; **(B)** Post-course minus pre-course mean differences.

### 3.3 Participants

Participants were vocational design students enrolled in the 72-h Furniture Appreciation and Selection course at Jinhua University of Vocational Technology, China, during the February–June 2026 teaching period. The study included only students aged 18 years or older. Convenience sampling was used because the study was conducted within an existing course. All students were informed that participation was voluntary and that their decision to participate or withdraw would not affect their course grades, teacher evaluation, or other academic rights.

The analytical export contained 150 consenting students with complete matched pre-course, post-course, and expert-rating records. All 150 records in the export were retained after screening; none was excluded for missing item responses, duplicate codes, unmatched records, age ineligibility, or out-of-range values. The archived analytical and consent materials did not contain a separate course-roster or invitation denominator beyond these 150 consenting matched records; therefore, an invited-to-enrolled attrition figure could not be reconstructed. The participants included 51 male students (34.0%), 97 female students (64.7%), and 2 students who preferred not to report their gender (1.3%). Their ages ranged from 18 to 23 years, with a mean age of 19.81 years (*SD* = 1.15). Most participants were second-year students (*n* = 104, 69.3%), followed by first-year students (*n* = 24, 16.0%) and third-year students (*n* = 22, 14.7%).

In terms of major, 63 students were from Environmental Art Design (42.0%), 45 from Interior Design (30.0%), 22 from Product Design (14.7%), 12 from Digital Media Design (8.0%), and 8 from other related design majors (5.3%). Before the course, 62 students (41.3%) reported no previous furniture-related learning experience, 72 students (48.0%) reported basic experience, and 16 students (10.7%) reported extensive experience. Regarding prior GenAI use, 28 students (18.7%) had never used GenAI tools, 83 students (55.3%) used them occasionally, and 39 students (26.0%) used them frequently.

Sample size was determined by the available eligible matched course cohort rather than an *a priori* power target. A sensitivity calculation at α = 0.05 and 80% power indicated that N = 150 could detect a two-sided correlation of |r| ≥ 0.226 and an overall regression effect of f^2^ ≥ 0.066 for a model with two predictors; smaller associations may have gone undetected.

Questionnaire data and expert-rated creative design performance scores were matched using anonymous student codes. No real names, student ID numbers, phone numbers, or other sensitive personal information were collected. The research team provided written study information and obtained written consent before questionnaire completion; signed consent records were maintained separately from the anonymous research data. The study was reviewed and approved by the Academic Ethics Committee of Jinhua University of Vocational Technology (Academic Ethics Review 2026 No. 014) before recruitment, consent, and data collection began. Because teacher blinding to participation status before final grade determination was not formally recorded, no such blinding is claimed.

### 3.4 Measures

#### 3.4.1 Construct specification and measurement-assessment strategy

The measurement strategy separates three questions that require separate consideration: what each construct is intended to represent, how the course-context items were developed or adapted, and whether the observed item responses support the intended factorial structure. AISL and SDC were specified as domain-specific single constructs, CSE as a single creative-confidence construct, and LE as a broad engagement construct whose cognitive, emotional, and behavioral content was examined empirically. Internal consistency is therefore treated as descriptive evidence rather than proof of dimensionality. Item-level confirmatory factor analysis (CFA) was conducted before the composite-score association analysis was interpreted. Because all questionnaire indicators had five ordered response categories and showed only mild univariate skewness and kurtosis in this sample, maximum-likelihood CFA was estimated from the item covariance matrix. The analysis was conducted on the validated *N* = 150 dataset using a Python/NumPy/SciPy implementation; the model specifications and comparison criteria are described in Sections 3.7.1 and 4.3 and in Table 2.

**Table 2:** CFA-based measurement diagnostics for the four post-course questionnaire constructs.

Construct	Items	Cronbach’s α	Std. loading range	CR	AVE
AISL	6	0.799	0.590–0.668	0.799	0.399
SDC	6	0.736	0.491–0.713	0.737	0.324
CSE	6	0.781	0.443–0.741	0.785	0.383
LE	12	0.827	0.452–0.644	0.828	0.288

Standardized loadings are from the four-factor CFA. CR, composite reliability; AVE, average variance extracted. CR exceeded 0.70 for all four constructs, whereas AVE remained below 0.50; convergent evidence is therefore interpreted as modest rather than unequivocal. The four-factor model nevertheless showed acceptable global fit and substantially outperformed the one-factor alternative. Table 2 reports the loading ranges and reliability diagnostics, and Section 4.3 reports the model-comparison statistics.

The questionnaire covered perceived GenAI-supported learning, sustainable design cognition, creative self-efficacy, and learning engagement. Items used a five-point scale from 1 = strongly disagree to 5 = strongly agree, and no item was reverse scored. Sustainable design cognition and creative self-efficacy were assessed before and after the course; perceived GenAI-supported learning and engagement were assessed after the course because they referred to the completed learning experience. Three experts reviewed the context-specific wording before administration to improve clarity and task relevance; this expert review is not presented as a formal content-validity index or as independent psychometric validation. The construct definitions, response scale, scoring approach, measurement timing, source attribution, and expert-rating criteria are described in Sections 3.4.2–3.5. Because several items were developed or adapted for this course, construct dimensionality is evaluated empirically in the analysis before composite scores are interpreted in the structural association model.

#### 3.4.2 Perceived GenAI-supported learning

Perceived GenAI-supported learning (AISL) was measured with six course-context items assessing students’ retrospective appraisal of how GenAI tools supported style understanding, idea expansion, scheme expression, learning efficiency, exploration of furniture combinations, and independent design thinking. AISL is deliberately defined as an appraisal construct. It does not measure the number of AI interactions, time on task, prompt quality, platform/model exposure, output quality, or dependence on generated content. Students with the same AISL score may therefore have engaged with GenAI in materially different ways. The construct is interpreted as perceived learning support rather than as an instructional dose or objective measure of GenAI use.

#### 3.4.3 Sustainable design cognition

Sustainable design cognition (SDC) was measured with six course-context items representing students’ application-oriented understanding of sustainable furniture and interior-design decisions. The construct was specified to cover environmental responsibility, resource conservation, durability, repairability, health and comfort, and long-term value rather than general environmental attitudes. The item domain was informed by design-for-sustainability and design-education literature (Ceschin and Gaziulusoy, 2016; Stegall, 2006; Boks and Diehl, 2006; Watkins et al., 2021; Faludi et al., 2023; Afacan, 2014; Al-Saud et al., 2024; Chellappa and Rohatgi, 2025). Because these items were developed for the present course, their internal consistency is not treated as sufficient evidence of construct validity; the analysis evaluates their factorial coherence explicitly.

#### 3.4.4 Creative self-efficacy

Creative self-efficacy (CSE) was measured with six items adapted to the furniture-design learning context from the established definition of belief in one’s capability to produce creative outcomes (Tierney and Farmer, 2002). Adaptation preserved the intended creative-confidence construct while placing the statements in course-relevant language. Because contextual adaptation can alter item functioning, CSE is included in the factorial assessment rather than being assumed to retain its original dimensional properties automatically.

#### 3.4.5 Learning engagement

Learning engagement (LE) was measured with 12 items covering cognitive, emotional, and behavioral engagement, with four items representing each content domain (Fredricks et al., 2004). The cognitive, emotional, and behavioral four-item subsets had alphas of 0.623, 0.561, and 0.583, respectively, whereas the 12-item score had α = 0.827. The analysis therefore did not infer a general engagement construct from the higher pooled alpha alone. A one-factor 12-item CFA showed good fit, χ^2^ (54) = 63.96, *p* = 0.166, CFI = 0.971, TLI = 0.965, RMSEA = 0.035, 90% CI [0.000, 0.065], SRMR = 0.058. A correlated three-factor model did not improve fit (χ^2^ (51) = 63.88, *p* = 0.106, CFI = 0.963, TLI = 0.952, RMSEA = 0.041, 90% CI [0.000, 0.070], SRMR = 0.058) and produced near-unity latent correlations (0.978–0.995). Information criteria also favored the simpler one-factor solution (AIC = 111.96 vs. 117.88; BIC = 184.21 vs. 199.16). On this basis, the broader 12-item LE score was retained as a parsimonious general engagement measure. The three short content-domain alphas remain reported transparently and are not interpreted as reliable stand-alone subscales.

### 3.5 Expert-rated creative design performance evaluation

Expert-rated creative design performance was evaluated from students’ final furniture selection and design projects by three independent experts using a five-point scale. The outcome is an applied design-performance construct rather than a context-free test of divergent creativity. Novelty represents the originality component of creativity, while usefulness and the remaining task-specific criteria represent effectiveness and appropriateness within the furniture-design brief. This operationalization follows the originality-effectiveness definition of creativity (Runco and Jaeger, 2012) while recognizing that performance in an applied design course also requires aesthetic, spatial, sustainability, and technical adequacy. Independent qualified judges were used in accordance with the consensual assessment tradition (Amabile, 1982).

The seven criteria were novelty, usefulness, aesthetic quality, spatial suitability, sustainability, technical presentation, and overall quality. For each student, the primary creative design performance score was calculated as the equal-weight mean of the 21 ratings provided by the three experts across the seven criteria. The first six criteria captured specific attributes of applied creative design performance. Overall quality was a holistic judgment of coherence, maturity, and completeness rather than a mathematical summary of the six preceding ratings. To test whether the main result depended on this global criterion, the analysis already included a six-criterion sensitivity outcome excluding overall quality (LE-CP: β = 0.499, *p* < 0.001, R^2^ = 0.249; six- versus seven-criterion score correlation r = 0.992).

A narrower sensitivity analysis used only novelty and usefulness, the two criteria most directly aligned with the conventional originality-effectiveness definition of creativity. The LE association remained positive and substantial (β = 0.474, SE = 0.072, t = 6.553, *p* < 0.001, R^2^ = 0.225, f^2^ = 0.290). The narrow two-criterion score correlated r = 0.899 with the seven-criterion creative design performance score. Thus, the central LE–performance result did not depend on inclusion of the broader aesthetic, spatial, sustainability, technical, or overall-quality criteria.

### 3.6 Data collection procedure

Data collection took place from February to June 2026 and proceeded in three stages. First, before course activities began, students completed the pre-course questionnaire. It collected demographic and learning-background information, including gender, age, grade level, major, prior furniture-related learning experience, prior GenAI use, sustainability knowledge, design competition or exhibition experience, and self-rated design ability. Sustainable design cognition and creative self-efficacy were also measured at this stage.

Second, after the 72-h course, students completed the post-course questionnaire, which measured perceived GenAI-supported learning, sustainable design cognition, creative self-efficacy, and learning engagement. Sustainable design cognition and creative self-efficacy were measured at both points to examine pre–post changes.

Third, after students submitted their final furniture selection and design projects, three experts independently evaluated the anonymized project files using a common creative design performance rating form. The evaluation covered seven dimensions: novelty, usefulness, aesthetic quality, spatial suitability, sustainability, technical presentation, and overall quality. The experts were not provided with students’ questionnaire responses or the study hypotheses, and ratings were completed independently. The ratings were not used as the direct basis for course grades. The archived protocol, however, does not establish that every expert was unaware of every student’s previous in-class performance or classroom interaction; complete blinding to prior classroom performance is therefore not claimed. Official course-grade scores were not included in the validated de-identified analytical dataset, and the variable ‘Grade’ in that dataset denotes academic year. A grade-expert-score correlation could therefore not be calculated from the locked research data without adding an external data source. Anonymous student codes linked the pre-course questionnaire, post-course questionnaire, and expert-rated creative design performance scores.

Questionnaire measures were collected at two points and the final design outcome was evaluated by independent experts, providing temporal separation for the pre-post measures and source separation for the final outcome. These procedures reduce exclusive reliance on one source, but they do not demonstrate that common-method variance is absent (Podsakoff et al., 2003). AISL, SDC, CSE, and LE were all self-reported at the same post-course occasion; therefore, shared respondent, response-style, and occasion effects remain plausible contributors to their covariance. The interpretation of H1-H4 and the coefficient products explicitly retains this limitation rather than using VIF or HTMT as tests of common-method bias. Figure 4 summarizes the timing of data collection, anonymous record linkage, and the two theory-specified analytical streams used in the study.

**Figure 4:**
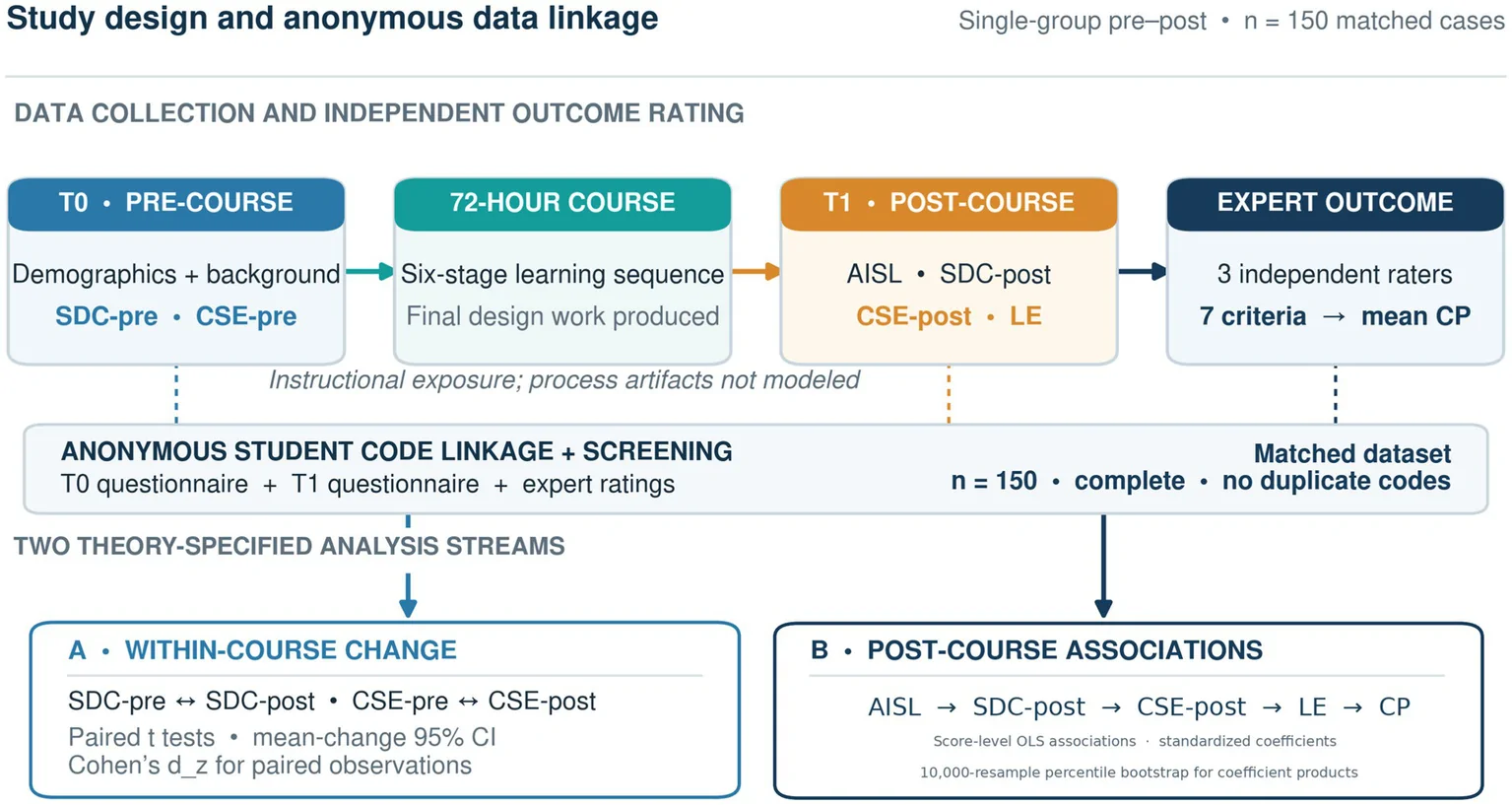
Study design and data linkage. The pre-course and post-course questionnaires and expert ratings were matched using anonymous student codes. Arrows indicate the actual sequence of data collection and record linkage; they do not establish temporal precedence, mediation, or causal direction among the contemporaneous post-course psychological variables.

### 3.7 Data analysis

All final analyses were conducted using the de-identified matched dataset. The score-level analyses were implemented in Python 3.12.13 (NumPy 2.3.5, pandas 2.2.3, SciPy 1.17.0, and openpyxl 3.1.5). An item-level measurement-model stage preceded substantive score-level interpretation. Screening covered missing item responses, duplicate anonymous codes, unmatched pre-course, post-course, and expert-rating records, age eligibility, and out-of-range values. The final analytical file contained 150 matched cases with no missing item values or duplicate codes. The scoring approach, measurement timing, expert-rating criteria, model specifications, bootstrap seed, and sensitivity-analysis definitions used in the reported analyses are described in the relevant Methods subsections. The author(s) declared that Generative AI was used in the creation of this manuscript. Generative AI was used only for language polishing, grammar checking, and improving the clarity of expression during manuscript preparation. It was not used for data collection, questionnaire administration, statistical analysis, interpretation of results, or replacement of authorial judgment. The author reviewed, verified, and revised all AI-assisted text and takes full responsibility for the final content of the manuscript.

#### 3.7.1 Item-level measurement-model assessment

Measurement adequacy was evaluated before the structural association analyses. A four-factor CFA specified AISL, SDC, CSE, and the 12-item general LE score as correlated latent constructs. Model evaluation used χ^2^, df, CFI, TLI, RMSEA with a 90% confidence interval, SRMR, standardized factor loadings, composite reliability (CR), average variance extracted (AVE), and latent-factor correlations. The theory-specified model was compared with a one-factor alternative in which all 30 questionnaire items loaded on one common factor. For LE specifically, a one-factor 12-item model was compared with a correlated three-factor model representing cognitive, emotional, and behavioral engagement. No item was deleted and no residual covariance was freed solely to improve model fit.

#### 3.7.2 Within-course change and score-level association analyses

Descriptive statistics were calculated for participant characteristics and focal scores. Equal-weight scores were means of their constituent items only after the relevant score definition had been examined in the measurement-model stage. Paired-samples t tests described within-course changes in SDC and CSE, with mean-change confidence intervals and Cohen’s d_z_. Pearson correlations summarized bivariate post-course relationships. These analyses are descriptive and associational; the pre-post comparisons are not interpreted as GenAI-specific effects.

The five direct hypotheses were estimated with ordinary least squares regressions of standardized construct scores: SDC on AISL; CSE jointly on AISL and SDC; LE on CSE; and expert-rated creative design performance on LE. Standardized coefficients (β), analytic standard errors, t values, *p* values, local f^2^, and R^2^ were reported. Composite-score OLS was retained as the primary substantive summary because the hypotheses concern transparent score-level associations and the final outcome is an observed expert-derived composite. However, averaging items does not remove measurement error; it leaves measurement error unmodeled. Accordingly, score-level OLS is interpreted only after factorial support for the score definitions has been established, and the coefficients are not presented as latent-variable structural estimates. Small coefficients are interpreted with particular caution.

Percentile confidence intervals were obtained from 10,000 nonparametric case-resampling bootstrap samples (seed 20,260,807). Direct coefficients and theory-specified coefficient products were recalculated in every sample. An interval excluding zero was described as statistically detectable, not as automatic substantive confirmation of a mechanism. Magnitude was evaluated separately, with particular attention to the four-coefficient H8 product. Because AISL, SDC, CSE, and LE were measured at the same post-course occasion, coefficient products summarize compatibility with the theory-specified ordering rather than temporal mediation.

#### 3.7.3 Measurement-error sensitivity analysis

To address the possibility that score averaging attenuates or otherwise alters associations among multi-item psychological constructs, a latent-variable sensitivity model was estimated after the measurement model had been evaluated. The structural ordering matched the score-level analysis, while AISL, SDC, CSE, and LE were represented by their item-level latent factors and the expert-rated CP composite remained an observed outcome. The model fit was acceptable for a sensitivity analysis, χ^2^ (430) = 505.09, *p* = 0.007, CFI = 0.930, TLI = 0.924, RMSEA = 0.034, SRMR = 0.078. The standardized latent structural estimates were 0.260 for AISL–SDC, 0.368 for AISL–CSE, 0.274 for SDC–CSE, 0.551 for CSE–LE, and 0.575 for LE–CP. All estimates remained positive and the LE–CP relationship remained the strongest. The latent model is used only as a robustness check; it does not alter the non-causal interpretation of the contemporaneous design.

#### 3.7.4 Measured-covariate sensitivity analyses

To examine whether the focal score-level associations were sensitive to measured background differences, each primary regression equation was re-estimated with gender and prior GenAI experience as covariates. Gender was represented by indicator variables with male as the reference category; prior GenAI use (never = 0, occasionally = 1, frequently = 2) was entered as a standardized ordered covariate. Because SDC and CSE were also measured before the course, additional baseline-augmented models included the corresponding pre-course score. These models are treated as robustness checks rather than causal adjustments, because measured covariate control cannot eliminate unmeasured confounding.

## 4 Results

### 4.1 Data screening and participant characteristics

Pre-course questionnaires, post-course questionnaires, and expert ratings were linked through anonymous student codes and screened for completeness, eligibility, range violations, and matching. The final analytic sample comprised 150 matched cases, with no missing values or duplicate codes.

Participants included 51 male students (34.0%), 97 female students (64.7%), and 2 students who preferred not to report gender (1.3%). Ages ranged from 18 to 23 years (*M* = 19.81, *SD* = 1.15). Most participants were second-year students (*n* = 104, 69.3%). Table 3 summarizes demographic and learning-background characteristics.

**Table 3:** Participant characteristics.

Variable	Category	*n*	%
Gender	Male	51	34.0
	Female	97	64.7
	Prefer not to answer	2	1.3
Grade	First year	24	16.0
	Second year	104	69.3
	Third year	22	14.7
Major	Environmental Art Design	63	42.0
	Interior Design	45	30.0
	Product Design	22	14.7
	Digital Media Design	12	8.0
	Other	8	5.3
Prior furniture-related learning experience	None	62	41.3
	Basic experience	72	48.0
	Extensive experience	16	10.7
Prior GenAI use	Never	28	18.7
	Occasionally	83	55.3
	Frequently	39	26.0
Prior design competition/exhibition experience	No	100	66.7
	Yes	50	33.3

### 4.2 Descriptive statistics and normality

Table 4 presents descriptive statistics. Skewness ranged from −0.403 to 0.069 and kurtosis from −0.336 to −0.035, indicating no marked univariate departure from normality.

**Table 4:** Descriptive statistics of focal variables.

Variable	Code	Mean	SD	Min	Max	Skewness	Kurtosis
Perceived GenAI-supported learning	AISL	4.117	0.526	2.500	5.000	−0.403	−0.182
Sustainable design cognition, pre-course	SDC_pre	3.326	0.615	2.000	4.833	−0.038	−0.328
Sustainable design cognition, post-course	SDC_post	3.792	0.476	2.500	4.833	−0.127	−0.336
Creative self-efficacy, pre-course	CSE_pre	3.509	0.628	1.833	5.000	−0.125	−0.223
Creative self-efficacy, post-course	CSE_post	3.877	0.510	2.500	5.000	−0.105	−0.277
Learning engagement	LE	3.612	0.428	2.417	4.667	0.069	−0.087
Creative design performance	CP	3.410	0.464	2.191	4.620	0.068	−0.035

### 4.3 Measurement-model assessment and score diagnostics

The four-factor CFA provided an acceptable, although not uniformly strong, representation of the 30 post-course questionnaire items: χ^2^ (399) = 464.28, *p* = 0.013, CFI = 0.936, TLI = 0.930, RMSEA = 0.033, 90% CI [0.016, 0.046], and SRMR = 0.070. The corresponding one-factor alternative fit substantially worse, χ^2^ (405) = 820.73, CFI = 0.593, TLI = 0.563, RMSEA = 0.083, and SRMR = 0.103; the difference in χ^2^ was 356.45 for 6 df (*p* < 0.001). Latent correlations in the four-factor model ranged from 0.259 to 0.489, which supports separation of AISL, SDC, CSE, and LE in this sample. Standardized item loadings ranged from 0.590 to 0.668 for AISL, 0.491 to 0.713 for SDC, 0.443 to 0.741 for CSE, and 0.452 to 0.644 for LE. CR values ranged from 0.737 to 0.828. AVE values were below 0.50 for all four constructs (0.399, 0.324, 0.383, and 0.288, respectively), indicating that convergent evidence remained modest rather than unequivocal. The scores were therefore retained for the association analyses, but small structural coefficients are interpreted cautiously and the measures are not presented as fully validated general-purpose scales.

For LE, the one-factor 12-item model fit the data well, χ^2^ (54) = 63.96, *p* = 0.166, CFI = 0.971, TLI = 0.965, RMSEA = 0.035, 90% CI [0.000, 0.065], SRMR = 0.058. The correlated three-factor model did not improve fit and produced latent correlations of 0.978–0.995 among cognitive, emotional, and behavioral engagement. Because the more complex model offered no empirical advantage and the three dimensions were nearly indistinguishable in this sample, the parsimonious 12-item general LE score was retained. This decision is based on factorial evidence rather than on the increase in Cronbach’s alpha after pooling the items. Table 2 summarizes the CFA-based measurement diagnostics for the four post-course questionnaire constructs.

### 4.4 Score collinearity and inter-rater reliability

Score-level VIF values ranged from 1.126 to 1.580, indicating no immediate collinearity problem among the focal scores. These values are not evidence that common-method dependence is negligible. AISL, SDC, CSE, and LE were all self-reported at the same post-course occasion, so shared respondent, response-style, and occasion effects may contribute to their covariance. The independent expert outcome provides source separation only for the final performance variable.

Under a two-way random-effects, absolute-agreement model, overall creative design performance showed ICC (2, 1) = 0.776 for a single expert and ICC (2, 3) = 0.912 for the mean of three experts (Koo and Li, 2016). Dimension-level ICC (2, 3) values ranged from 0.637 to 0.741. Reliability was therefore excellent for the mean overall score used in the analysis and more moderate for individual dimensions.

Outcome-definition sensitivity analyses were also consistent with H5. Excluding overall quality yielded β = 0.499, *p* < 0.001, R^2^ = 0.249, and using only novelty and usefulness yielded β = 0.474, SE = 0.072, *p* < 0.001, R^2^ = 0.225, f^2^ = 0.290. The narrow originality-effectiveness score correlated r = 0.899 with the seven-criterion outcome. These checks indicate that the LE–performance association was not created by the global overall-quality criterion or by the broader design-performance definition.

### 4.5 Pre-course and post-course changes

Paired-samples *t* tests showed significant within-course increases in sustainable design cognition, from 3.326 to 3.792 (mean change = 0.467, 95% CI [0.378, 0.556]), *t*(149) = 10.362, *p* < 0.001, *d_z_* = 0.846, and creative self-efficacy, from 3.509 to 3.877 (mean change = 0.368, 95% CI [0.270, 0.466]), *t*(149) = 7.424, *p* < 0.001, *d_z_* = 0.606 (Table 5; Figure 5). Without a comparison group, these increases cannot be attributed specifically to GenAI-supported activities.

**Table 5:** Within-course changes in sustainable design cognition and creative self-efficacy.

Variable	Pre-course Mean ± SD	Post-course Mean ± SD	Mean difference	t	df	*p*	Cohen’s d_z_
Sustainable design cognition	3.326 ± 0.615	3.792 ± 0.476	0.467	10.362	149	< 0.001	0.846
Creative self-efficacy	3.509 ± 0.628	3.877 ± 0.510	0.368	7.424	149	< 0.001	0.606

**Figure 5:**
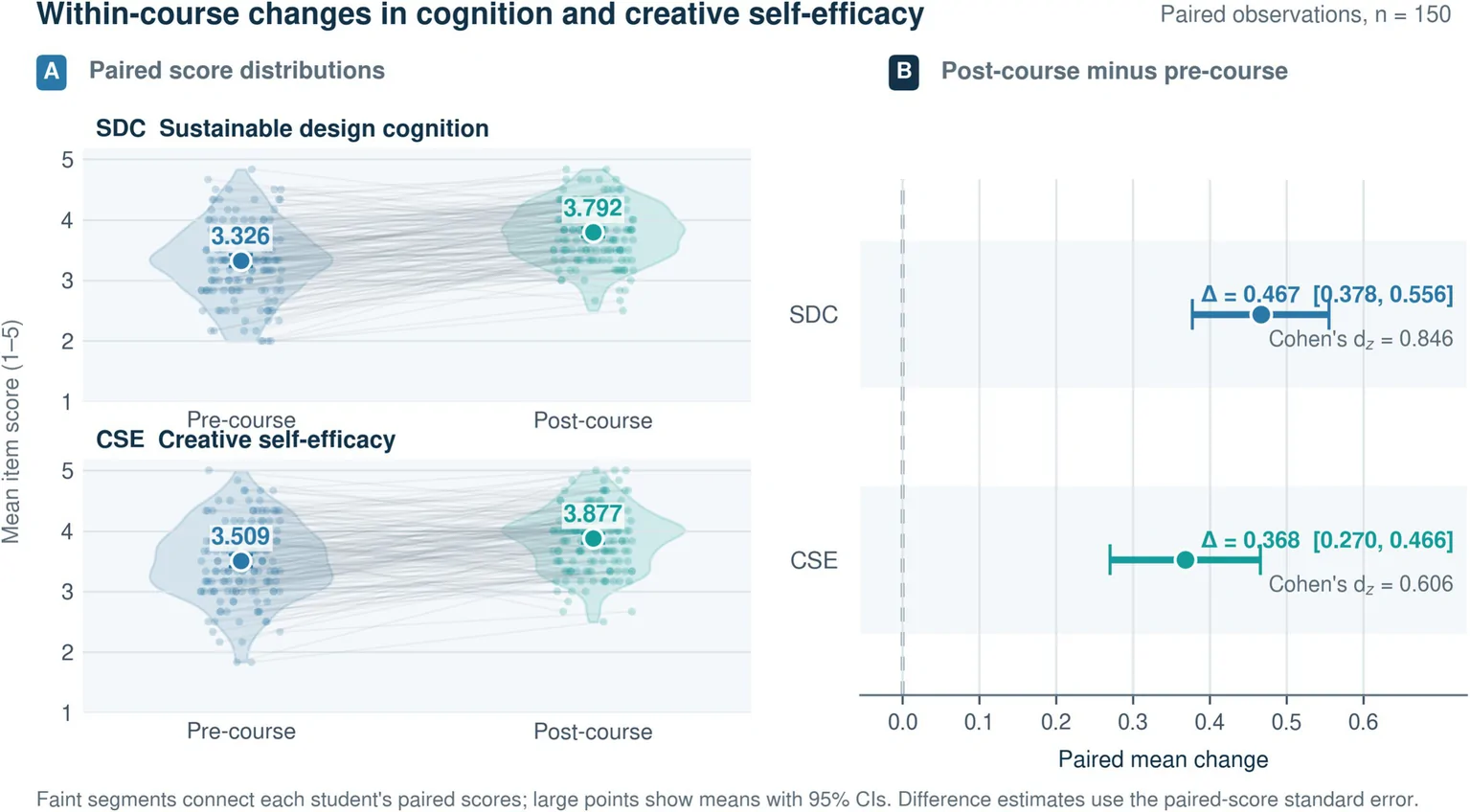
Within-course changes in sustainable design cognition and creative self-efficacy (*N* = 150). Left panels show paired student observations and group means with 95% confidence intervals; Right panels show the mean Post − Pre difference with its 95% confidence interval and paired-samples *dz*.

### 4.6 Correlation analysis

All focal variables were positively correlated. Learning engagement showed the strongest bivariate association with expert-rated creative design performance (r = 0.515, *p* < 0.001). Score-level VIF values ranged from 1.126 to 1.580 and did not indicate an immediate collinearity problem (Section 4.4).

### 4.7 Theory-specified score-level associations

Table 1 and Figure 6 summarize the theory-specified post-course score-level associations. AISL was positively associated with SDC (β = 0.204, SE = 0.080, *p* = 0.012; f^2^ = 0.044) and CSE (β = 0.298, SE = 0.078, *p* < 0.001; f^2^ = 0.100). SDC was positively associated with CSE (β = 0.192, SE = 0.078, *p* = 0.015; f^2^ = 0.041). CSE was positively associated with LE (β = 0.396, SE = 0.075, *p* < 0.001; f^2^ = 0.186), and LE was positively associated with expert-rated creative design performance (β = 0.515, SE = 0.070, *p* < 0.001; f^2^ = 0.361). Thus, all five direct associations were statistically detectable in the hypothesized positive direction, but their substantive magnitudes differed considerably.

**Figure 6:**
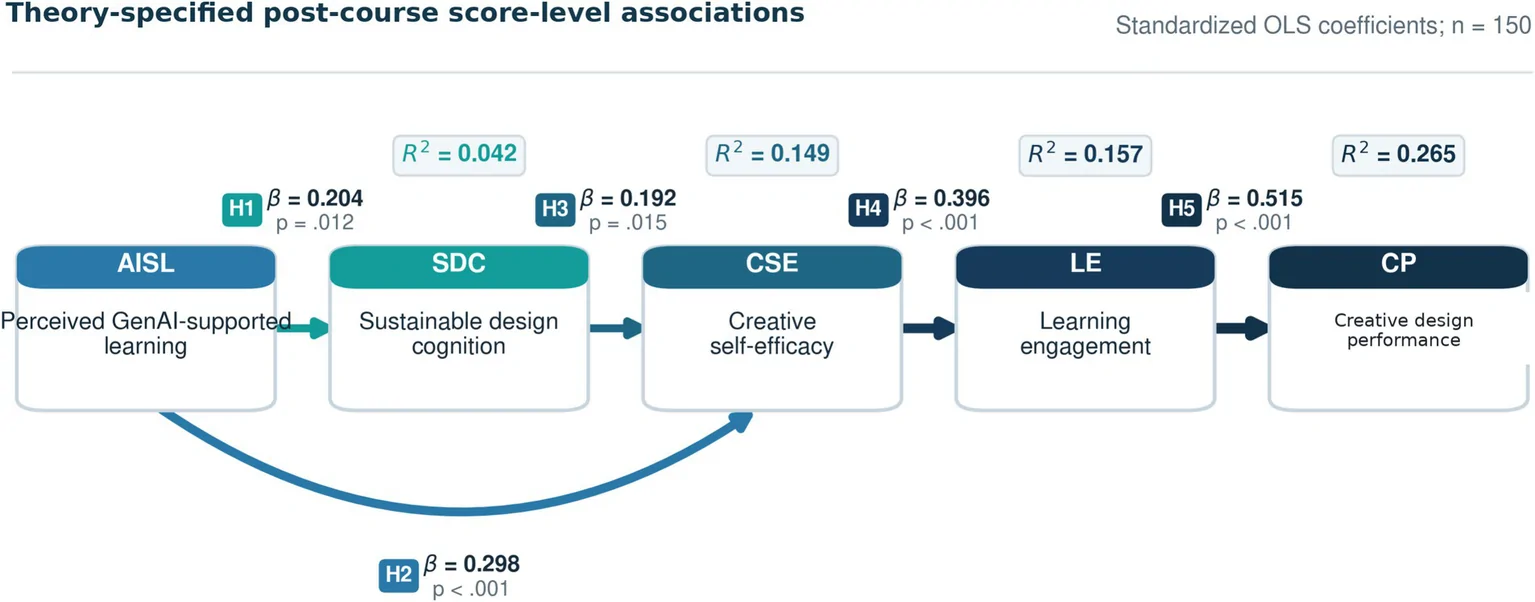
Theory-specified post-course score-level associations. Standardized OLS coefficients and analytic *p* values are shown for each association; *R*^2^ is reported for outcome scores. Arrows indicate analytical ordering only and do not establish temporal precedence, mediation, or causal direction.

The standardized OLS equations explained 4.2% of the variance in SDC, 14.9% in CSE, 15.7% in LE, and 26.5% in creative design performance. The effect-size gradient is central to the interpretation: AISL-SDC and SDC-CSE had small local effects, AISL-CSE was modest, CSE-LE was larger, and LE-CP was the strongest association and local contribution. The manuscript therefore does not treat five statistically detectable coefficients as equally strong confirmation of one complete sequence.

The latent-variable sensitivity model produced the same directional pattern and preserved the relative prominence of the later associations. Standardized latent estimates were 0.260 (AISL–SDC), 0.368 (AISL–CSE), 0.274 (SDC–CSE), 0.551 (CSE–LE), and 0.575 (LE–CP). Thus, modeling measurement error did not reverse the substantive ordering observed in the composite-score analysis, although the latent estimates are not used to strengthen causal claims.

### 4.8 Theory-specified coefficient products

The SDC-CSE-LE coefficient product was 0.076 (bootstrap SE = 0.037, 95% CI [0.0114, 0.1574]). The interval excluded zero, indicating that the theory-specified product was statistically detectable. Because SDC, CSE, and LE were not measured in temporally ordered waves, this result is not described as evidence that CSE mediated a temporal effect of SDC on engagement.

The CSE-LE-CP coefficient product was 0.204 (bootstrap SE = 0.051, 95% CI [0.1095, 0.3089]). This product was statistically detectable and substantially larger than the SDC-CSE-LE and full four-coefficient products. It is interpreted as compatibility with the specified contemporaneous ordering, not as a confirmed mediation mechanism.

The four-coefficient AISL-SDC-CSE-LE-CP product was 0.0080 (bootstrap SE = 0.0060), with an unrounded 95% CI of [0.0002, 0.0234]. The interval narrowly excluded zero, so the product was statistically detectable. Its magnitude, however, was negligible. H8 is therefore not treated as substantive confirmation of a complete psychological mechanism; it is reported as a near-zero coefficient product that sets an empirical boundary on the proposed ordering. Figure 2 visualizes the three theory-specified coefficient products and their percentile 95% confidence intervals.

The H6-H8 coefficient products were statistically compatible with the specified positive ordering, but their magnitudes differed markedly. None is interpreted as a causal mediation effect because the constituent questionnaire constructs were not measured at distinct temporally ordered waves and were not experimentally manipulated.

### 4.9 Measured-covariate sensitivity analyses

With gender and prior GenAI experience included as measured covariates, the focal coefficients were 0.189 for AISL-SDC (SE = 0.096, *p* = 0.051), 0.318 for AISL-CSE (SE = 0.092, *p* < 0.001), 0.200 for SDC-CSE (SE = 0.079, *p* = 0.012), 0.378 for CSE-LE (SE = 0.076, *p* < 0.001), and 0.525 for LE-CP (SE = 0.072, *p* < 0.001). The direction and approximate magnitude of all five associations remained stable, although AISL-SDC became borderline under this background-adjusted specification. In a baseline-augmented model that also controlled pre-course SDC, AISL-SDCpost was 0.205 (*p* = 0.013). In the corresponding CSEpost model including pre-course CSE, AISL-CSE was 0.277 (*p* = 0.001) and SDC-CSE was 0.172 (*p* = 0.017). Together, these analyses support the overall directional pattern while showing that the modest AISL-SDC association is somewhat specification-sensitive.

## 5 Discussion

### 5.1 Principal findings

The matched pre-post results showed increases in SDC and CSE during the course, but these changes describe the course as a whole and cannot identify a GenAI-specific contribution. In the contemporaneous post-course association model, the clearest empirical pattern was not uniform support for a complete sequence. Rather, effect magnitudes increased toward the more proximal engagement-performance relationship: AISL-SDC and SDC-CSE were small, AISL-CSE was modest, CSE-LE was larger, and LE-CP was the strongest direct association (β = 0.515; f^2^ = 0.361). The full four-coefficient product was only 0.0080 and is now interpreted as negligible despite its bootstrap interval narrowly excluding zero.

This pattern is compatible with the analytical ordering shown in the framework, but it does not establish that the constructs unfolded in that sequence. AISL, SDC, CSE, and LE were measured at the same post-course occasion, and reverse or reciprocal explanations remain plausible. Social cognitive theory is therefore used to separate environmental appraisal, domain cognition, efficacy beliefs, engagement, and performance, while the data indicate how strongly those constructs co-varied in this course.

Keeping perceived support separate from evaluated performance is central to the interpretation. Students’ reports that GenAI helped them understand or develop ideas were not counted as evidence of a better final product. Experts evaluated the completed work, and engagement—not perceived GenAI support—was the theory-specified variable directly associated with that outcome. The practical value of GenAI may therefore depend less on its presence than on whether students use it to compare, question, revise, and justify their design decisions.

### 5.2 Within-course changes in sustainable design cognition and creative self-efficacy

For RQ1, sustainable design cognition showed a large within-course increase (*d_z_* = 0.846), and creative self-efficacy showed a moderate increase (*d_z_* = 0.606). Sustainability was addressed through material analysis, furniture selection, spatial configuration, durability, repairability, health, comfort, resource use, and final presentation. It functioned as a set of design criteria rather than an isolated topic. This approach is consistent with calls to embed sustainability in regular design decisions across the curriculum (Boks and Diehl, 2006; Watkins et al., 2021; Faludi et al., 2023).

The change in sustainable design cognition may reflect repeated opportunities to connect sustainability principles with concrete furniture decisions, but the present design cannot attribute that change to GenAI. Sustainability instruction, task repetition, teacher guidance, peer interaction, project practice, and increasing familiarity with furniture criteria all changed concurrently. Generated representations were one component of the learning environment, not an isolated treatment.

The increase in creative self-efficacy is also theoretically plausible. The course required students to externalize ideas, compare alternatives, revise generated content, respond to feedback, and present completed proposals. Such activities may provide experiences of task progress and successful problem resolution that are relevant to efficacy judgments (Bandura, 1997). The result is broadly consistent with Hwang and Wu (2025), who reported statistical associations connecting AI-integrated design education, self-efficacy, anxiety reduction, and creative cognition. It also converges with Song and Xu (2026), whose comparative study found higher creative self-efficacy under GenAI-supported learning combined with a structured design-thinking strategy.

The present findings nevertheless provide a different and more limited form of evidence. Song and Xu (2026) compared three instructional conditions, whereas the current study observed one cohort across one course. The present pre-course and post-course changes consequently indicate that cognition and confidence increased during the course, but they do not isolate which course component produced those changes. Teacher feedback, repeated measurement, maturation, concurrent learning, or students’ increasing familiarity with furniture design tasks remain plausible alternative explanations.

### 5.3 Direct contemporaneous associations among perceived GenAI support, cognition, self-efficacy, engagement, and performance

In response to RQ2, perceived GenAI-supported learning was positively associated with post-course sustainable design cognition, supporting H1. The association was statistically significant but modest (*β* = 0.204), and the model explained only 4.2% of the variance in sustainable design cognition. This result is educationally meaningful precisely because it does not suggest that students’ perceptions of GenAI support account for most of their sustainability understanding. Sustainable design cognition is likely to depend on broader instructional inputs, including explicit sustainability content, project criteria, teacher explanation, prior design knowledge, and opportunities to apply sustainability principles. GenAI may provide additional representations and comparisons, but visual generation alone is insufficient for developing sustainability-related judgment.

The measured-covariate sensitivity analysis reinforces the need for a cautious interpretation of H1. The AISL-SDC coefficient remained similar in magnitude after adjustment for gender and prior GenAI experience (β = 0.189) but became borderline (*p* = 0.051); when pre-course SDC was also included, the AISL-SDCpost coefficient was 0.205 (*p* = 0.013). Thus, the observed association is modest and somewhat specification-sensitive rather than a dominant or invariant relationship.

AISL was positively associated with CSE (β = 0.298). Students who retrospectively judged GenAI as more supportive also tended to report stronger creative confidence. Because AISL is an appraisal rather than an exposure measure, the association should not be read as evidence that using more GenAI produces greater self-efficacy. Reciprocal interpretation is also plausible: students with stronger confidence, greater task success, or more sustained participation may be more likely to evaluate GenAI support positively.

The positive association between sustainable design cognition and creative self-efficacy (*β* = 0.192) supported H3. Although the effect was small, it suggests that domain-specific understanding may provide students with a basis for evaluating and defending creative decisions. Sustainability criteria can constrain some options, but such constraints need not suppress creativity. They may instead give students clearer grounds for distinguishing responsible proposals from visually attractive but impractical or environmentally superficial ones. In this sense, sustainable design cognition may function as an evaluative resource that supports purposeful creativity.

Creative self-efficacy was positively associated with learning engagement (*β* = 0.396), supporting H4. This result accords with social cognitive theory, which connects efficacy beliefs with task choice, effort, persistence, and responses to difficulty. In design learning, students who are more confident in their creative capability may be more willing to examine alternatives, tolerate incomplete early ideas, request feedback, and continue revising. Nevertheless, because creative self-efficacy and engagement were measured at the same post-course point, the analysis cannot exclude reciprocal relationships. Engagement may also provide successful learning experiences that strengthen creative self-efficacy.

Learning engagement showed the strongest bivariate association with expert-rated creative design performance and the largest coefficient among the five theory-specified direct associations (β = 0.515), supporting H5. This finding is consistent with Qian et al. (2026), who connected learning engagement with creativity performance in a course-based design context. It also clarifies why GenAI access alone is unlikely to guarantee a high-quality outcome. Furniture design work requires sustained problem interpretation, comparison of materials and spatial relationships, response to feedback, technical refinement, and coherent presentation. Engagement captures participation in these activities and is therefore more closely connected with completed design quality than students’ general perception that GenAI supported their learning.

The prominence of engagement also places the study within a wider educational-psychology perspective on GenAI. Huang et al. (2026) showed that the learning value of AI-related support cues depends partly on whether the resulting experience is cognitively manageable, rather than on maximizing interaction. Similarly, Simo and Lina (2026) identified heterogeneous profiles of AI literacy and cognitive flexibility associated with different levels of deep learning. Although cognitive load, AI literacy, and cognitive flexibility were not measured in the present study and should not be inserted retrospectively into its model, these findings reinforce the broader interpretation that students do not benefit uniformly from AI-supported environments. Perceived support must still be converted into cognitively active and sustained participation.

### 5.4 Coefficient products and the limits of analytical ordering

For RQ3, the SDC-CSE-LE coefficient product was 0.076 and its bootstrap interval excluded zero. This result shows that the two positive score-level associations yield a positive product in the specified ordering. It does not establish that SDC changed CSE before CSE changed engagement.

The CSE-LE-CP product was larger (0.204). This result is substantively more prominent because the CSE-LE association was moderate and the LE-CP association was the strongest direct relationship in the model. Even so, CSE and LE were assessed at the same post-course occasion, so the product is not interpreted as a verified temporal mediation process.

The coefficient products are descriptive summaries of theory-specified contemporaneous associations, not confirmed mediation mechanisms. Bootstrap confidence intervals indicate sampling uncertainty around the products; they do not create temporal precedence that the design did not observe. Because AISL, SDC, CSE, and LE were reported by the same students at the same post-course occasion, part of the covariance represented in these coefficient products may also reflect shared respondent and measurement-occasion effects.

The full AISL-SDC-CSE-LE-CP product was 0.0080. Its unrounded 95% bootstrap interval [0.0002, 0.0234] narrowly excluded zero, but the value was negligible. We therefore do not classify H8 as substantive confirmation of a complete psychological sequence. Instead, H8 is treated as a boundary-setting result showing that a statistically detectable four-coefficient product can carry almost no practical weight.

The negligible H8 magnitude argues strongly against treating perceived GenAI support as the dominant starting point of creative design performance. The present data support a much stronger statement about the proximal engagement-performance association than about a distal technology-to-performance chain. Other factors such as prior design competence, feedback, peer interaction, task difficulty, and the quality of actual GenAI use may provide alternative routes or boundary conditions, but they were not tested here.

A conditional interpretation is consistent with evidence that GenAI-supported learning can involve both helpful and harmful processes. Xiaohui and Khan (2026), for example, linked AI dependency with lower academic motivation through learned helplessness and reported a moderating role for AI literacy. Their variables differ from those used here, but their findings reinforce a basic point: perceived educational support is not the same as unrestricted or dependent use. GenAI is most defensible when it is embedded in a structured learning process and remains subject to student judgment.

### 5.5 Theoretical, context-specific, and practical contributions

The study makes a domain-specific theoretical contribution by bringing sustainable design cognition into research on GenAI-supported learning. Treating this construct as a professional judgment resource clarifies how students may evaluate generated alternatives against durability, repairability, resource use, health, comfort, and long-term value. It also extends a literature that has focused more often on creative cognition, AI acceptance, cognitive load, dependency, intention, or deep learning.

The analytical contribution lies in separating perceived technological support, domain cognition, creative confidence, engagement, and evaluated performance while assigning different theoretical weight to relationships of different magnitude. The strongest evidence concerns the proximal LE-CP association; the earlier AISL/SDC links are modest, and the full four-coefficient product is negligible. The contribution is therefore not that eight statistically detectable results validate an entire psychological chain. Rather, the model shows that distinct educational-psychology constructs can be separated empirically and that their observed relationships differ sharply in substantive strength.

The model is therefore more informative as a map of differentiated associations than as evidence for a single sequential psychological process. This distinction allows the theoretical interpretation to follow the observed gradient of effect magnitude rather than the number of statistically detectable hypotheses.

The outcome evidence provides a further contribution, but the construct is described more precisely as expert-rated creative design performance. The seven-criterion rating combines originality with task-specific effectiveness and design-quality criteria. This broader operationalization supports ecological validity in a furniture-design course, but it should not be confused with a pure divergent-thinking score. Two sensitivity analyses help define the boundary of the outcome: removing overall quality left the LE association almost unchanged (β = 0.499), and restricting the outcome to novelty and usefulness still produced a substantial association (β = 0.474, R^2^ = 0.225). The narrow score also correlated r = 0.899 with the seven-criterion score. The central engagement-performance finding was therefore robust to narrower definitions of creativity, while the broader label ‘creative design performance’ remains the most accurate description of the primary outcome.

The study provides context-specific evidence from an underrepresented Chinese vocational design setting. It does not claim that the observed coefficients are internationally generalizable. What may be transferable is the analytical distinction among perceived technological support, disciplinary cognition, creative confidence, engagement, and independently evaluated work, together with the instructional emphasis on comparison, reflection, revision, and disciplinary judgment. Cross-institutional and cross-national replication is required before broader measurement or effect equivalence can be claimed.

What can transfer across national settings is the analytical and instructional logic, not a particular platform or an assumed effect size. Other vocational and practice-based programs can test whether defining criteria, generating alternatives, comparing outputs, revising with disciplinary knowledge, and justifying final decisions produce similar patterns. Cross-national research should examine measurement equivalence and whether engagement remains the most proximal correlate of independently assessed performance.

For teaching, increasing GenAI use is not an adequate objective. The technology should be placed inside a design process that preserves student judgment and responsibility. Before generation, students need to define users, spatial constraints, and sustainability criteria. During generation, they should retain prompts, compare alternatives, identify inaccuracies, and explain why an output is accepted or rejected. Afterwards, they should revise the proposal with disciplinary knowledge and show how durability, repairability, health, comfort, resource use, and long-term value are addressed.

This interpretation is close to Song and Xu (2026), who found better outcomes when GenAI was combined with structured design thinking. It is also consistent with recent design-education evidence: Hatef Jalil and Abdullah (2026) embedded GenAI within a scaffolded design-thinking model in vocational fashion design, and Balasubramanian (2026) emphasized curriculum integration in which GenAI supports rather than replaces human creative judgment. It also fits Huang et al. (2026), whose results caution against maximizing interaction when cognitive burden increases. A staged sequence—problem definition, sustainability evaluation, alternative generation, critical comparison, revision, and final justification—is more educationally defensible than unrestricted production of images or text.

Engagement deserves particular attention because it showed the strongest bivariate association with expert-rated performance and the largest coefficient among the theory-specified direct associations. Staged submissions, critiques, feedback cycles, comparison tasks, and documented revisions make the learning process visible and keep students responsible for the work. Guidance may also need to differ across students. Simo and Lina (2026) found heterogeneous profiles of AI literacy and cognitive flexibility, although neither construct was measured here. Future studies should test, rather than assume, whether these characteristics alter the conversion of perceived support into sustained engagement.

The practical message is straightforward: GenAI can scaffold comparison, reflection, and iteration, but it cannot substitute for sustainable judgment or creative agency. In this dataset, performance was related most strongly to students’ engagement in analysis, feedback, revision, and presentation—not simply to their perception that the technology was helpful.

### 5.6 Limitations and future research

Several limitations define what can be concluded. First, one cohort was followed through one 72-h course without a comparison group. The increases in sustainable design cognition and creative self-efficacy occurred during the course, but they cannot be attributed specifically to GenAI-supported activities. Sustainability instruction, teacher guidance, project practice, peer interaction, repeated measurement, and familiarity with the tasks developed over the same period. Comparative, randomized, or carefully matched designs are needed to isolate the contribution of particular instructional components.

Second, most variables in the post-course association model were measured after the course. The directions were specified theoretically, but the data do not establish temporal precedence among sustainable design cognition, creative self-efficacy, and engagement. This limitation applies to H6, H7, and H8 even though their bootstrap intervals excluded zero. Longitudinal or cross-lagged designs with measurements before instruction, during project development, after the course, and at follow-up would be better suited to testing reciprocal or temporally ordered mechanisms.

Third, AISL, SDC, CSE, and LE were self-reported, and the four post-course questionnaire constructs share the same respondent and measurement occasion. Common-method dependence, social-desirability responses, and response-style effects therefore cannot be ruled out. The four-factor CFA supported separation of the constructs at the global-model level and clearly outperformed a one-factor alternative, but standardized loadings were moderate and AVE remained below 0.50 for AISL, SDC, CSE, and LE. Thus, the CFA provides within-sample factorial evidence rather than full independent validation. The latent-variable sensitivity analysis preserved the direction and relative pattern of the score-level associations, but replication in independent samples, measurement-invariance testing, and additional behavioral or observational indicators remain necessary.

Fourth, three experts supplied an outcome independent of the questionnaires, but the seven-criterion composite represents applied creative design performance rather than a context-free measure of creativity alone. Aesthetic quality, spatial suitability, sustainability, technical presentation, and overall quality broaden the construct beyond originality and usefulness. The six-criterion and narrow originality-effectiveness sensitivity analyses are therefore important for defining what is robust across outcome operationalizations. Repeated ratings of idea generation, alternative evaluation, prototype development, and final presentation would further clarify where creative quality emerges. In addition, complete blinding of the experts to students’ prior classroom performance was not documented, and official course-grade scores were not available in the locked analytical dataset for an expert-score/grade comparison. Potential rater-context bias therefore cannot be ruled out and should be addressed prospectively in future studies.

Fifth, perceived GenAI-supported learning was an appraisal, not an objective record of frequency, quality, or independence of use. Tool and model versions were neither standardized nor recorded at the participant level. Students with similar perception scores may therefore differ in platform access, prompting, critical evaluation, revision, reliance on generated content, and integration of disciplinary knowledge. Prompt histories, output comparisons, design journals, revision logs, screen-capture records, and platform telemetry are needed to identify which forms of GenAI-supported activity promote reflective development and which encourage superficial adoption.

Finally, the sample came from one vocational design course and one institutional setting. Generalization to other disciplines, education levels, institutions, or cultures should be cautious. The sample-size sensitivity analysis indicated 80% power to detect correlations of approximately |*r*| ≥ 0.226, but smaller effects and complex subgroup or conditional patterns may have gone undetected. Larger multi-institutional studies could test the model across different design programs, levels of prior experience, and types of GenAI-supported task.

## 6 Conclusion

This within-course correlational study examined change during a 72-h GenAI-supported vocational furniture course and contemporaneous post-course associations among perceived GenAI support, sustainable design cognition, creative self-efficacy, learning engagement, and expert-rated creative design performance. SDC and CSE increased during the course, but those changes cannot be attributed specifically to GenAI. After the course, perceived GenAI support co-varied with SDC and CSE, CSE co-varied with engagement, and engagement showed the strongest direct association with expert-rated creative design performance. These relationships are theory-organized score-level associations, not verified temporal pathways.

The three theory-specified coefficient products were positive, but their magnitudes were not equally informative. The CSE-LE-CP product was substantially larger than the full AISL-SDC-CSE-LE-CP product, and H8 was negligible despite its confidence interval narrowly excluding zero. The conclusion therefore distinguishes statistical detectability from substantive support and does not use H8 as evidence for a meaningful causal or serial psychological mechanism.

The study adds context-specific evidence from one Chinese vocational design course. Its central contribution is to separate perceived technological support from the cognitive, motivational, and behavioral processes associated with independently evaluated work. The analytical and instructional logic may be tested in other vocational and practice-based programs, but the present coefficients, measurement properties, and platform-related effects should not be assumed to generalize across institutions, disciplines, or countries. Cross-institutional and cross-national replication is required before broader applicability can be claimed.

## Data Availability

The raw data supporting the conclusions of this article will be made available by the authors, without undue reservation.
