# Supplementary material for: Psychological associations between perceived generative AI-supported learning and creative design performance in vocational design education: a within-course correlational study
Source: Front Psychol. 2026 Oct 7;17:1962146. doi: 10.3389/fpsyg.2026.1962146 (PMC13683594; doi:10.3389/fpsyg.2026.1962146)
Supplement: Supplementary file 1 [file Supplementary_file_1.docx]

**Supplementary Material**

Supplementary Table S1. Course design for the 72-hour Furniture Appreciation and Selection course.

| **Stage** | **Hours** | **Teaching focus** | **Learning activities** | **Role of GenAI** | **Student outputs** | **Related variables** |
| --- | --- | --- | --- | --- | --- | --- |
| Stage 1 | 12 | Furniture appreciation and style recognition | Case analysis; style comparison | Visual references; style-board support | Style analysis card; comparison card | SDC; LE |
| Stage 2 | 12 | Materials, craftsmanship, and structure | Material analysis; structural disassembly | Material-case organization; process visualization | Material sheet; structural analysis | SDC; CSE |
| Stage 3 | 12 | Furniture selection and spatial configuration | User analysis; furniture selection | User-profile support; atmosphere references | Selection scheme; spatial layout | LE |
| Stage 4 | 12 | Sustainable furniture design cognition | Sustainable case analysis; green-material comparison | Sustainable-case search; strategy generation | Sustainability strategy sheet | SDC |
| Stage 5 | 16 | GenAI-supported furniture concept generation | Prompt training; concept generation; iteration | Style board; material board; concept generation | GenAI-use record; concept drafts | AISL; CSE; LE |
| Stage 6 | 8 | Final presentation and evaluation | Presentation; peer feedback; expert rating | Presentation optimization | Final design work; expert rating | LE; CP |

Note. GenAI outputs served as references for comparison, ideation, and visualization; they were not accepted as final submissions and were not analyzed as separate research variables.

Supplementary Table S2. Complete construct items and expert-rating criteria.

| **Construct** | **Code** | **Full English wording / criterion** | **Administration or source** |
| --- | --- | --- | --- |
| Perceived GenAI-supported learning | AISL1 | AIGC tools helped me understand furniture styles more clearly. AIGC工具帮助我更清楚地理解家具风格。 | Post-course questionnaire |
| Perceived GenAI-supported learning | AISL2 | AIGC tools helped me expand my ideas for furniture selection or design. AIGC工具帮助我拓展家具选配或设计思路。 | Post-course questionnaire |
| Perceived GenAI-supported learning | AISL3 | AIGC tools helped me compare different furniture styles, materials, or spatial effects. AIGC工具帮助我比较不同的家具风格、材料或空间效果。 | Post-course questionnaire |
| Perceived GenAI-supported learning | AISL4 | AIGC tools improved the efficiency of my design expression. AIGC工具提高了我的设计表达效率。 | Post-course questionnaire |
| Perceived GenAI-supported learning | AISL5 | AIGC tools helped me revise and improve my furniture scheme. AIGC工具帮助我修改和完善家具方案。 | Post-course questionnaire |
| Perceived GenAI-supported learning | AISL6 | AIGC tools stimulated my independent thinking rather than replacing my design work. AIGC工具激发了我的独立思考，而不是替代我完成设计。 | Post-course questionnaire |
| Sustainable design cognition | SDC1 | I understand the relationship between sustainable design and furniture design. 我理解可持续设计与家具设计之间的关系。 | Pre- and post-course questionnaire |
| Sustainable design cognition | SDC2 | I can identify environmentally friendly or recyclable furniture materials. 我能够识别环保或可回收家具材料。 | Pre- and post-course questionnaire |
| Sustainable design cognition | SDC3 | I consider durability and long-term use value when evaluating furniture. 我评价家具时会考虑耐用性和长期使用价值。 | Pre- and post-course questionnaire |
| Sustainable design cognition | SDC4 | I understand that furniture design should consider users’ health and comfort. 我认为家具设计应关注用户健康和舒适体验。 | Pre- and post-course questionnaire |
| Sustainable design cognition | SDC5 | I can consider resource conservation and waste reduction in furniture selection. 我能够从节约资源和减少浪费角度进行家具选配。 | Pre- and post-course questionnaire |
| Sustainable design cognition | SDC6 | I can apply sustainable design principles to furniture selection or design solutions. 我能够将可持续设计理念应用于家具选配或设计方案。 | Pre- and post-course questionnaire |
| Creative self-efficacy | CSE1 | I believe I can develop creative furniture design ideas. 我相信自己能够提出具有创意的家具设计想法。 | Pre- and post-course questionnaire |
| Creative self-efficacy | CSE2 | I am confident in solving furniture style, material, and spatial problems. 我有信心解决家具风格、材料和空间搭配问题。 | Pre- and post-course questionnaire |
| Creative self-efficacy | CSE3 | I can transform daily-life needs into design ideas. 我能够将生活需求转化为设计想法。 | Pre- and post-course questionnaire |
| Creative self-efficacy | CSE4 | I can improve my design work through repeated modification. 我能够通过不断修改完善设计作品。 | Pre- and post-course questionnaire |
| Creative self-efficacy | CSE5 | I believe I can complete furniture-related creative tasks successfully. 我相信自己能够完成家具相关创意任务。 | Pre- and post-course questionnaire |
| Creative self-efficacy | CSE6 | I have confidence in presenting my own design concepts. 我有信心表达自己的设计理念。 | Pre- and post-course questionnaire |
| Cognitive engagement | CE1 | I actively analyzed furniture styles, materials, and spatial relationships during the course. 课程中，我主动分析家具风格、材料和空间关系。 | Post-course questionnaire |
| Cognitive engagement | CE2 | I tried to connect furniture appreciation knowledge with real design problems. 我尝试将家具欣赏知识与真实设计问题联系起来。 | Post-course questionnaire |
| Cognitive engagement | CE3 | I compared different design possibilities before making my final decision. 在确定最终方案前，我比较了不同的设计可能性。 | Post-course questionnaire |
| Cognitive engagement | CE4 | I reflected on how sustainability could be applied to furniture selection or design. 我思考了如何将可持续理念应用于家具选配或设计。 | Post-course questionnaire |
| Emotional engagement | EE1 | I was interested in the learning tasks of this course. 我对本课程的学习任务感兴趣。 | Post-course questionnaire |
| Emotional engagement | EE2 | I felt a sense of achievement when improving my furniture scheme. 在完善家具方案时，我感到有成就感。 | Post-course questionnaire |
| Emotional engagement | EE3 | I became more willing to learn about furniture appreciation and selection. 我更加愿意学习家具欣赏与选配相关内容。 | Post-course questionnaire |
| Emotional engagement | EE4 | I felt motivated when using AI tools to explore design possibilities. 使用AI工具探索设计可能性时，我感到有学习动力。 | Post-course questionnaire |
| Behavioral engagement | BE1 | I completed the course tasks carefully. 我认真完成了课程任务。 | Post-course questionnaire |
| Behavioral engagement | BE2 | I searched for additional references to improve my furniture scheme. 我主动查找资料来完善家具方案。 | Post-course questionnaire |
| Behavioral engagement | BE3 | I revised my scheme repeatedly based on feedback. 我根据反馈多次修改方案。 | Post-course questionnaire |
| Behavioral engagement | BE4 | I actively participated in discussion, presentation, or peer feedback activities. 我积极参与讨论、汇报或同伴反馈活动。 | Post-course questionnaire |
| Creative design performance | CP1 | Novelty: the work demonstrates originality and avoids simple imitation or template-based expression. | Independent expert rating of final work |
| Creative design performance | CP2 | Usefulness: the work responds to real user needs and presents a reasonable functional arrangement. | Independent expert rating of final work |
| Creative design performance | CP3 | Aesthetic quality: furniture style, color, materials, and spatial atmosphere are coordinated. | Independent expert rating of final work |
| Creative design performance | CP4 | Spatial suitability: furniture scale, spatial function, circulation, and use context are appropriately matched. | Independent expert rating of final work |
| Creative design performance | CP5 | Sustainability: the work addresses environmentally preferable materials, durability, maintainability, low-carbon strategies, or waste reduction. | Independent expert rating of final work |
| Creative design performance | CP6 | Technical presentation: drawings, renderings, material specifications, GenAI-use records, and design explanations are clear and complete. | Independent expert rating of final work |
| Creative design performance | CP7 | Overall quality: the work shows coherent overall logic, visual communication, design maturity, and presentation completeness. | Independent expert rating of final work |

Note. Questionnaire items used a five-point scale from 1 = strongly disagree to 5 = strongly agree. Expert criteria used a five-point scale from 1 = very weak to 5 = excellent. No item was reverse scored. Measurement bases: AISL, Huang et al. (2024) and Habib et al. (2024); SDC, Afacan (2014), Ceschin and Gaziulusoy (2016), Faludi et al. (2023), and Al-Saud et al. (2024); CSE, Tierney and Farmer (2002) and Beghetto (2006); LE, Fredricks et al. (2004), Reeve and Tseng (2011), and Qian et al. (2026); CP, Amabile (1982), Runco and Jaeger (2012), and Qian et al. (2026). Items were context-adapted and reviewed by three experts before administration.

Supplementary Table S3. Theory-specified hypotheses.

| **Hypothesis** | **Path** | **Statement** |
| --- | --- | --- |
| H1 | AISL → SDC | Perceived GenAI-supported learning is positively associated with post-course sustainable design cognition. |
| H2 | AISL → CSE | Perceived GenAI-supported learning is positively associated with post-course creative self-efficacy. |
| H3 | SDC → CSE | Post-course sustainable design cognition is positively associated with post-course creative self-efficacy. |
| H4 | CSE → LE | Post-course creative self-efficacy is positively associated with post-course learning engagement. |
| H5 | LE → CP | Post-course learning engagement is positively associated with expert-rated creative design performance. |
| H6 | SDC → CSE → LE | The theory-specified product of the post-course SDC-CSE and CSE-LE coefficients is positive. |
| H7 | CSE → LE → CP | The theory-specified product of the post-course CSE-LE and LE-CP coefficients is positive. |
| H8 | AISL → SDC → CSE → LE → CP | The theory-specified product of the post-course AISL-SDC, SDC-CSE, CSE-LE, and LE-CP coefficients is positive. |

Supplementary Table S4. Additional measurement, inter-rater, robustness, and sample-size diagnostics.

Panel A. Heterotrait–monotrait ratio (HTMT) matrix

| **Construct** | **AISL** | **SDC** | **CSE** | **LE** | **CP** |
| --- | --- | --- | --- | --- | --- |
| AISL | 1.000 | 0.287 | 0.429 | 0.442 | 0.256 |
| SDC | 0.287 | 1.000 | 0.345 | 0.352 | 0.276 |
| CSE | 0.429 | 0.345 | 1.000 | 0.487 | 0.434 |
| LE | 0.442 | 0.352 | 0.487 | 1.000 | 0.585 |
| CP | 0.256 | 0.276 | 0.434 | 0.585 | 1.000 |

Note. Maximum HTMT = 0.585; all pairwise values were below 0.85. AISL, SDC, and CSE were represented by their six individual item scores; LE was represented by the cognitive-, emotional-, and behavioral-engagement dimension means; CP was represented by seven expert-averaged rating dimensions.

Panel B. Inter-rater reliability from a two-way random-effects, absolute-agreement model

| **Rating dimension** | **ICC(2,3)** |
| --- | --- |
| Novelty | 0.741 |
| Usefulness | 0.688 |
| Aesthetic quality | 0.721 |
| Spatial suitability | 0.708 |
| Sustainability | 0.637 |
| Technical presentation | 0.665 |
| Overall quality | 0.662 |
| Overall creative design performance | 0.912 |

Note. ICC(2,3) estimates the reliability of the mean of three independent experts. Overall ICC(2,1) = 0.776 and ICC(2,3) = 0.912.

Panel C. Sensitivity analysis excluding the holistic overall-quality criterion

| **Creative design performance outcome** | **β (LE → CP)** | **p** | **R²** | **f²** | **Interpretation** |
| --- | --- | --- | --- | --- | --- |
| Primary seven-criterion score | 0.515 | < 0.001 | 0.265 | 0.361 | Primary analysis |
| Six domain-specific criteria; overall quality excluded | 0.499 | < 0.001 | 0.249 | 0.332 | Conclusion unchanged |

Note. The six-criterion and seven-criterion creative design performance scores correlated at r = 0.992. Removing overall quality did not materially change H5.

Panel D. Sample-size sensitivity

| **N** | **α** | **Power** | **Analysis** | **Smallest detectable effect** |
| --- | --- | --- | --- | --- |
| 150 | 0.05 | 0.80 | Two-sided Pearson correlation | \|r\| = 0.226 |
| 150 | 0.05 | 0.80 | Overall F test with two predictors | f² = 0.066 |

Note. Sensitivity values describe detectable effects for the available course cohort and do not represent an a priori sample-size calculation.
